# Modulation of Protein Fermentation Does Not Affect Fecal Water Toxicity: A Randomized Cross-Over Study in Healthy Subjects

**DOI:** 10.1371/journal.pone.0052387

**Published:** 2012-12-20

**Authors:** Karen Windey, Vicky De Preter, Thierry Louat, Frans Schuit, Jean Herman, Greet Vansant, Kristin Verbeke

**Affiliations:** 1 Translational Research Center for Gastrointestinal Disorders and Leuven Food Science and Nutrition Research Centre, University Hospital Gasthuisberg, KU Leven Leuven, Belgium; 2 Interface Valorisation Platform, KU Leven Leuven, Belgium; 3 Department of Nutrition–Public Health Medicine, Leuven Food Science and Nutrition Research Centre, KU Leven Leuven, Belgium; 4 Gene Expression Unit, Department of Molecular Cell Biology, KU Leven, Leuven, Belgium; Paris Institute of Technology for Life, Food and Environmental Sciences, France

## Abstract

**Objective:**

Protein fermentation results in production of metabolites such as ammonia, amines and indolic, phenolic and sulfur-containing compounds. *In vitro* studies suggest that these metabolites might be toxic. However, human and animal studies do not consistently support these findings. We modified protein fermentation in healthy subjects to assess the effects on colonic metabolism and parameters of gut health, and to identify metabolites associated with toxicity.

**Design:**

After a 2-week run-in period with normal protein intake (NP), 20 healthy subjects followed an isocaloric high protein (HP) and low protein (LP) diet for 2 weeks in a cross-over design. Protein fermentation was estimated from urinary p-cresol excretion. Fecal metabolite profiles were analyzed using GC-MS and compared using cluster analysis. DGGE was used to analyze microbiota composition. Fecal water genotoxicity and cytotoxicity were determined using the Comet assay and the WST-1-assay, respectively, and were related to the metabolite profiles.

**Results:**

Dietary protein intake was significantly higher during the HP diet compared to the NP and LP diet. Urinary p-cresol excretion correlated positively with protein intake. Fecal water cytotoxicity correlated negatively with protein fermentation, while fecal water genotoxicity was not correlated with protein fermentation. Heptanal, 3-methyl-2-butanone, dimethyl disulfide and 2-propenyl ester of acetic acid are associated with genotoxicity and indole, 1-octanol, heptanal, 2,4-dithiapentane, allyl-isothiocyanate, 1-methyl-4-(1-methylethenyl)-benzene, propionic acid, octanoic acid, nonanoic acid and decanoic acid with cytotoxicity.

**Conclusion:**

This study does not support a role of protein fermentation in gut toxicity. The identified metabolites can provide new insight into colonic health.

**Trial Registration:**

ClinicalTrial.gov NCT01280513

## Introduction

Protein fermentation is widely recognized to be detrimental to gut health. Protein fermentation or putrefaction is the anaerobic digestion of protein by the microbiota residing in the colon. Proteins entering the colon originate from dietary proteins that escaped digestion in the proximal gut, pancreatic or intestinal secretions or desquamated gut cells. Increased protein intake results in increased protein fermentation in both animals and humans [1], [2]. Protein fermentation results in the production of branched chain fatty acids (BCFA; isobutyric and isovaleric acid) and short chain fatty acids (SCFA; acetic acid, propionic acid and butyric acid) but also of metabolites such as ammonia (NH_3_), amines, indolic, phenolic and sulfur-containing compounds [3].

Several *in vitro* studies investigated the effects of protein fermentation metabolites in relation to gut toxicity. Ammonia was found to increase cell proliferation and decrease cell permeability in colonic adenocarcinoma cells (CaCo-2) at concentrations between 10 and 100 mM [4], [5]. Also phenol (1–10 mmol/L) increases cell permeability [6]. Attene-Ramos *et al.* showed that hydrogen sulfide (H_2_S; 250 µmol/L) induces genotoxic damage in colonic adenocarcinoma cells (HT-29) [7] and suggests that this could be radical-mediated [8]. H_2_S also impairs butyrate oxidation, which is the most important energy pathway in colonocytes [9], [10].

Additional evidence on protein fermentation toxicity arises from animal studies. In a study where rats received a high casein diet (25%) urinary levels of p-cresol, a marker of protein fermentation, significantly correlated with genetic damage, indicating a possible role of protein fermentation in genotoxicity [2]. Genotoxicity of fecal water also increased in rats on a high soya diet (25%). Unfortunately, no markers of protein fermentation were included in this study [11]. In contrast, when casein that was thermolyzed for different times to make it less digestible was administered to rats to increase protein fermentation no association was found between formation of aberrant crypt foci and protein fermentation in rats [12].

Several epidemiological studies found an association between meat intake and both colorectal cancer (CRC) and inflammatory bowel disease (IBD). However, those studies focused on meat intake [13]–[16] or protein intake [17] but not on protein fermentation. As high meat intake is not only associated with higher protein intake but also with a higher intake of fat [14], heterocyclic amines [18] and heme [19], it is extremely difficult to attribute the increased CRC and IBD risk to protein intake or fermentation. In addition, this association was not found in all epidemiological studies [20], [21].

In the present human intervention study, we specifically modified the degree of protein fermentation by changing protein intake and evaluated the impact on parameters of gut health by investigating fecal water genotoxicity and cytotoxicity. A metabolome approach was applied to identify those metabolites associated with increased genotoxicity and cytotoxicity.

## Materials and Methods

### Study Population

**Figure 1:**
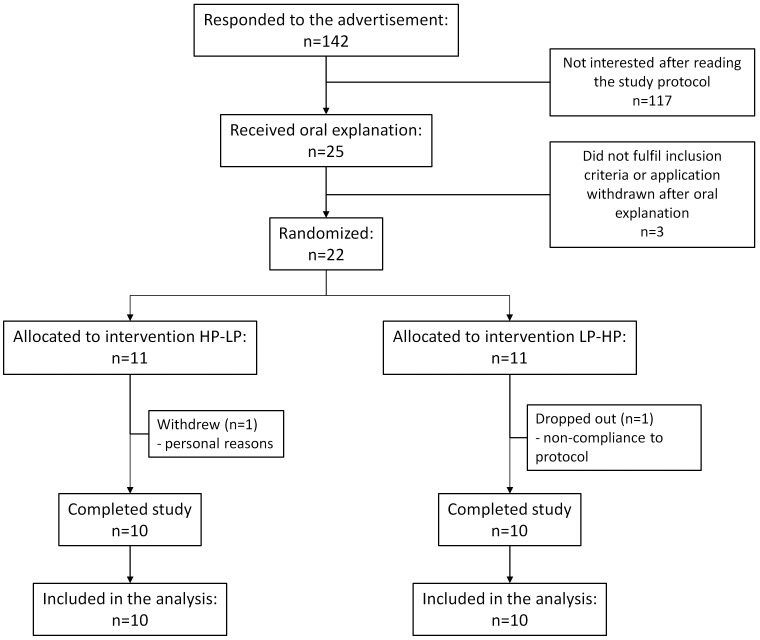
Enrollment of the volunteers.

Based on results from a pilot study in 9 subjects, a sample size of 18 healthy subjects was expected to provide 80% chance for detecting a difference of 15% (SD = 10.05) in fecal water genotoxicity (the primary outcome variable of this study) between the high and low protein diet at the 5% level of significance. Participants were recruited by advertisement among the students of the Catholic University of Leuven and among employees of the University Hospitals Leuven. Twenty-two healthy subjects with a regular dietary pattern (3 meals per day on at least 5 days per week) participated in the study between October 2009 and June 2010 (Figure 1). Exclusion criteria were abdominal surgery in the past (except from appendectomy), liver- or kidney failure, history of chronic gastro-intestinal conditions such as IBD, irritable bowel syndrome and celiac disease. Also subjects that consulted a dietician in the 6 weeks prior to the start of the trial or that were on a low-calorie or vegetarian diet were excluded. Female subjects were excluded if pregnant or lactating. All subjects were free of medication influencing the gut transit or intestinal microbiota for 14 days and of antibiotics for 1 month before the start of the trial. Intake of pre- and probiotics was prohibited during the complete study period. Twenty subjects (14 women, 6 men, age range: 19–41 years, BMI range: 18.5–25.5 kg/m^2^) completed the study. At the time of inclusion, the subjects were informed about pre- and probiotics and the foods containing them. The study was conducted according to the guidelines laid down in the Declaration of Helsinki and was approved by The Ethics Committee of the University Hospitals Leuven. The trial was registered at ClinicalTrial.gov (clinical trial number: NCT01280513). All subjects gave their written informed consent before participation. The protocol for this study and supporting CONSORT checklist are available as supporting information; Protocol S1 and Checklist S1.

### Study Design

The study started with a two-weeks run-in period during which the subjects consumed their habitual normal protein diet (NP) and was followed by two consecutive treatment periods (each two weeks) during which the subjects consumed either a high protein diet (HP, >25% energy derived from protein intake) or a low protein diet (LP, 9% of energy derived from protein intake), yet not necessarily in this order. The first week of each intervention was considered as an adaption period to the new diet. All dietary information and samples were collected during the second week of the intervention. After providing informed consent, the eligible subjects (n = 22) were randomly assigned to one of the randomization groups differing in the sequence of the intervention diets. Randomization and enrollment of the subjects was performed by an independent researcher who is no part of the study using online randomization software (www.randomizer.org). The type of intervention made it impossible to blind the treatment for both the subjects and the researchers.

During the first week of the run-in period, the subjects completed a 7-day dietary record. Subjects were asked to record all food and beverage intake, including weights and brands. All subjects received a kitchen scale to be able to quantify their dietary intake. In total, there were 3 clinical visits during the study. On the morning of day 5 subjects came to the Laboratory Absorption & Digestion from the Catholic University Leuven, Belgium, after a night of starvation. All subjects underwent a ventilated-hood calorimetry [22] for the assessment of their caloric needs and completed the Baecke questionnaire for physical activity [23]. They were weighed and performed a test during which they provided baseline samples, consumed a test meal and collected samples until the morning of day 7 (see below).

During the second week of the run-in period, subjects continued their habitual diet. This period was used to draw up individually adapted diets for each dietary period. Adapted diets were isocaloric to the habitual diet of the subjects and took the personal preferences of the subjects into account. Protein was replaced by digestible carbohydrates and fat and fiber intake were kept as constant as possible during the intervention. At the start of the first intervention period, each subject received two series (one for the LP diet and one for the HP diet) of 3 lists with 7 suggestions for breakfast, lunch and dinner, respectively, and 1 list with 10 possible snacks. They were allowed to choose one breakfast, one lunch and one dinner suggestion per day. Depending on their caloric needs, subjects were allowed to consume two or three snacks per day. The HP diet was supplemented with 20 g protein powder/day (Resource, Nestlé Healthcare Nutrition, Switzerland). During the second week of each dietary period the subjects completed a 7-day dietary record to evaluate the actual food intake. After the first dietary period subjects immediately switched diets and started the second dietary period. On day 26 and on day 40 the subjects came to the laboratory for the second and third clinical visit, respectively. They were weighed and consumed a test meal and collected samples as after the 1^st^ visit.

### Study End Points

The primary outcome variable of this study was fecal water genotoxicity. Secondary outcome variables included fecal water cytotoxicity, urinary p-cresol excretion, dietary composition, energy need and intake, changes in metabolite profiles, ammonia metabolism and microbiota composition.

### Ventilated-hood Calorimetry

The resting energy expenditure (REE) of the subjects was measured with indirect calorimetry [22], performed using a Datex calorimeter (Acertys Healthcare, Aartselaar, Belgium). The calorimeter was calibrated before each measurement. After an overnight fast, subjects came to the laboratory of the department of Nutrition-Public Health from the Catholic University of Leuven, Belgium, by bus or by car to avoid physical activity. In this way, they were in a metabolic state in which only fat oxidation occurs. They were put in a resting position (laying on a bed). A ventilated hood was positioned over the subject’s head and 40l of air/min was sucked out of the hood. After passage through a mixing chamber, the calorimeter calculated the amount of expired CO_2_ (VCO_2_) and inspired O_2_ (VO_2_) per minute, taking the composition of the surrounding air into account. Based on these data the REE was calculated each minute of the test using the Weir formula: REE (kcal/24 h) = 1.44×(3.9×VO_2_+1.1×VCO_2_) [24]. The entire measurement took 25 min and the average REE was calculated using the data of the last 15 min. The results were expressed as kcal/24 h.

### Test Meal

The test meal consisted of a pancake (8.4 g proteins, 11.2 g fat and 26.7 g carbohydrates; 244 kcal), labeled with lactose-[^15^N-^15^N]-ureide (75 mg) to evaluate the colonic ammonia metabolism. The latter substrate was synthesized according to the method of Schoorl as modified by Hofmann with [^15^N-^15^N]-urea obtained from Euriso-top (St-Aubin, Cédex, France) [25], [26]. To correct for differences in gastro-intestinal transit time 185 kBq of ^3^H-polyethylene glycol (^3^H-PEG; NEN Life Science Products Inc., Boston, MA, USA) was added to the test meal as an inert radiolabeled transit marker [27].

### Sample Collection

Before consuming the test meal a basal urine sample was collected. After intake of the test meal, urine was collected for 48 h in recipients containing neomycin to prevent bacterial growth. After measurement of the volumes, aliquots of urinary samples (40 mL) were stored at −20°C for further analysis. Fecal samples were collected for 72 h and delivered to the laboratory within 12 h. Upon delivery, 10 g of each sample was aliquoted for analysis of volatile organic compounds (VOC) and denaturing gradient gel electrophoresis (DGGE). Twenty grams of feces was centrifuged at 50 000×g at 4°C for 2 h for the production of fecal water. The remaining portions of the fecal samples were weighed, combined, and homogenized. Aliquots of these homogenized samples were lyophilized. Both wet and dry weights were measured to allow calculation of fecal dry weight (expressed as %). The fecal samples, fecal water and lyophilized fecal material were stored at −20°C.

### Analytical Procedures

#### Analysis of dietary intake

To assess actual dietary intake 7-day dietary records were analyzed using the online food calculator ‘Nubel’ (www.nubel.be) and information on standardized quantification of food products [28]. All dietary records were processed by the same person. Dietary analyses yielded information on average daily caloric intake (kcal), average intake on carbohydrates (g), proteins (g), fat (g) and fiber (g), and calcium (mg). Results are expressed as median (IQR).

#### Analysis of creatinine and urea

To assess the completeness of the urine collections, the ratio of observed to calculated creatinine excretion was calculated as proposed by Knuiman et al [29]. If this ratio was lower than 0.7, the collection was considered incomplete and the corresponding data were omitted from statistical analysis. Urea was measured in urine as a biomarker for protein intake. Creatinine and urea in urine were quantified using standard laboratory techniques.

#### Analysis of total N content and ^15^N-enrichment in urine and feces

Total N content and ^15^N-enrichment of urine and feces were measured using a continuous flow isotope ratio mass spectrometer coupled to an elemental analyzer (ANCA-2020, Europa Scientific, Crewe, UK). Urine (15 µL), absorbed on Chromosorb (Elemental Microanalysis Limited, Hampton, Devon, UK) or lyophilized feces (5–7 mg) were introduced into a combustion module for oxidation of N-compounds to nitrous oxides (N_x_O_y_) in the presence of copper oxide, O_2_ and chromium oxide at 1000°C. Subsequently, N_x_O_y_ were reduced using copper at 600°C to N_2_. The resulting gas was lead into the ion source of an isotope ratio mass spectrometer for the measurement of total N content and ^15^N-enrichment. Callisto CF-IRMS software (Version 8.0.41 (05), Sercon) was used for automatization of the elemental analyzer and for data acquisition. Results were expressed g/24 h (total N) or as percentage ^15^N of administered dose. Results are expressed as median (IQR).

#### Analysis of p-cresol in urine

Total p-cresol was measured in urine samples using gas chromatography-mass spectrometry (GC-MS type quadrupole; Trace GC-MS, Thermofinnigan, San José, CA, USA) [30]. Briefly, 50 µL p-cresol-d8 solution (200 mg/L) was added to 950 µl urine as internal standard. After adding 50 µL concentrated H_2_SO_4_, the samples were heated for 30 min at 90°C to deproteinize and hydrolyze the conjugated phenolic compounds. After cooling down to room temperature, p-cresol was extracted into 1 mL ethyl acetate. The ethyl acetate layer was dried using anhydrous Na_2_SO_4_ and finally 0.5 µL was analyzed on a GC-MS. Helium (high purity (>99.99%)) was used as a carrier gas with a constant flow of 1.3 mL/min. The analytical column was a Rxi-5ms (30 m×0.32 mm I.D., 1 µm film thickness, Restek, Bellefonte, PA, USA). The oven’s starting temperature was 55°C for 5 min and increased with 10°C/min to 160°C and with 20°C/min to 280°C (isothermal for 1 min). Mass spectrometric detection was performed in ‘single-ion-mass’ mode for masses m/z 107 (p-cresol) and m/z 114 (p-cresol-d8) with 2 scans/s. Results were expressed as mg p-cresol/24 h. XCalibur™ software (Version 1.4 SR1, Thermo Electron) was used for automatization of the GC-MS and for data acquisition.

#### Analysis of metabolite profiles in feces

VOC were analyzed using a purge-and-trap system (Velocity, Teldyne Tekmar, Mason, OH, USA) coupled on-line to a GC-MS type time of flight (GC-TOF-MS; Trace GC Thermoquest, Rodano, Italy and Tempus II, Thermo Electron, San José, CA, USA) [31]. A known amount of feces (0.25 mg) was suspended in 4870 µl H_2_O. A magnetic stirrer, a pinch of Na_2_SO_4_ to salt out the solution, 130 µL of pure H_2_SO_4_ and 130 µL internal standard (2-ethylbutyrate (20 mg/L), diethyl sulfide (0.25 mg/L) and 2,6-dimethylphenol (2.5 mg/L)) were added to each sample. Briefly, the VOC were purged out of the sample with a helium flow (high purity (>99.99%)) at a rate of 40 mL/min for 20 min at 70°C. Consequently, He was carried over a ‘dry flow’ column (Trap Tenax, Velocity, Interscience, Louvain-la-Neuve, Belgium) to control moisture transfer and VOC were concentrated on a second polar trap column (Trap Vocarb, Velocity, Interscience, Louvain-la-Neuve, Belgium). By raising the temperature to 250°C the VOC were desorbed from the column to the injector of the GC, where they are separated on an analytical column, AT Aquawax DA (30 m×0.25 mm I.D., 0.25 µm film thickness, Grace, Deerfield, IL, USA). The oven starting temperature was 35°C for 2 min and increased with 5°C/min to 100°C and with 10°C/min to 240°C. The final temperature was held constant for 5 min. Masses between m/z 30 and m/z 500 were detected with in full scan mode at 2 scans/s. XCalibur™ software (Version 1.4 SR1, Thermo Electron) was used for automatization of the GC-MS and for data acquisition.

The obtained chromatograms were processed using AMDIS (Automatic Mass Spectral Deconvolution and Identification Software version 2.1) provided by the US National Institute of Standards and Technology (NIST, Gaithersburg, MD, USA). This software provides quality matching using advanced spectral algorithms, adjacent peak deconvolution and background subtraction, which enables an unambiguous identification together with a quantitative indication of the metabolite levels. Identification of the metabolites in the samples was achieved by comparing the mass spectra of unknown peaks with the NIST library. Compounds showing mass spectra with match factors ≥90% were positively identified. Each sample was analyzed in duplicate. Relative indices of all VOC versus 2-ethylbutyrate as internal standard were calculated. A number of VOC were selected as markers for saccharolytic fermentation (acetic acid, propionic acid and butyric acid) and proteolytic fermentation (isobutyric acid, isovaleric acid, dimethyl sulfide and p-cresol). They were absolutely quantified with appropriate calibration curves obtained using internal standard quantification. The SCFA and BCFA were quantified using 2-ethylbutyrate as internal standard, whereas p-cresol and indole were quantified versus 2,6-dimethylphenol and dimethyl sulfide versus diethyl sulfide respectively. Results were expressed as mmol/L.

#### Analysis of ^3^H in lyophilized feces

The ^3^H-PEG content in 100 mg lyophilized fecal sample was measured with liquid scintillation counting (Packard Tricarb Liquid Scintillation Spectrometer, model 3375, Packard Instruments Inc., Downers Grove, IL, USA) after oxidation to ^3^H-H_2_O (Packard Sample Oxidizer, model 306, Packard Instruments Inc.). The ^3^H content in the fecal samples was expressed as percentage of administered dose recovered over 72 h and was used to correct ^15^N data for gastrointestinal transit by dividing the cumulative percentage of administered dose of ^15^N in fecal samples recovered over 72 h by the cumulative percentage of administered dose of ^3^H recovered over 72 h.

#### Analysis of the gut microbiota composition using DGGE

Total bacterial DNA was extracted from the fecal samples using a slightly modified version of the method of Pitcher et al [32], [33]. Briefly, a fecal sample suspension was made by homogenizing 0.50 mg fecal sample in PBS buffer (1% peptone). Of this suspension, 1 ml was centrifuged for 10 min at 13790×g. After removal of the supernatant, the pellet was resuspended in 1 ml TE buffer (10 mM Tris–HCl, 1 mM EDTA, pH 8.0) and centrifuged for 5 min at 13790×g. The pellet was resuspended in 150 µl enzyme solution (6 mg lysozyme powder and 40 µl mutanolysine dissolved in 110 µl TE buffer per sample) and incubated at 37°C for 40 min. Next, 500 µl GES solution (Guanidiumthiocyanate–EDTA–Sarkosyl; 600 g guanidiumthiocyanate, 200 ml 0.5 M EDTA, 10 g sarkosyl) was added and the samples were put on ice for 10 min. Then 250 µl cooled NH4Ac was added. After mixing the samples, they were put on ice for 10 min. Subsequently, 2 extractions were performed with chloroform/iso-amylalcohol (24/1). Then 700 µl supernatant was transferred to 378 µl isopropanol, mixed and centrifuged for 8 min at 51010×g. After removal of the supernatant, 150 µl ethanol (70%) was added and the samples were centrifuged for 2 min at 51010×g.After removal of the supernatant, the samples put in the vacuum centrifuge for 5 min. Then, 150 µl TE buffer was added to the DNA extract. Samples were stored at 4°C overnight. Finally, to remove residual RNA 1.5 µl RNAse solution was added and heated for 90 min at 37°C. DNA extracts were stored at −80°C. Next, community PCR was performed using universal primers F357+GC clamp and R518 targeting the hypervariable region of the 16S rRNA gene of bacteria. The resulting 16S rRNA gene amplicons were analyzed with DGGE using a 35–70% denaturating gradient as previously described resulting in profiles of the predominant fecal microbiota per subject [33]. On each DGGE gel, a standard reference consisting of an amplicon mix of 12 different bacterial species was included in the middle and at both exterior ends to allow digital gel normalization and comparison between gels. DGGE profiles were digitally processed with Bionumerics version 6.6 (Applied Maths, St-Martens-Latem, Belgium). After normalization of the gels, individual bands in each sample lane were marked using the auto search function of the software, followed by manual correction if necessary. All profiles were compared using the band matching tool and uncertain bands were excluded. Every band in a profile was allocated to its nearest band-class after a collective analysis of all profiles, in which common bands were traced across different sample profiles. A maximum error of 0.5% of deviation was applied, which means that a band was only allocated if it was located at a distance of less than 0.5% of the total length of the profile from the closest band-class. The designation of the band-classes was based on their position on the profile compared to the standard reference. The intensity of a given band-class was expressed in respect of the other band-classes on the same profile.

#### Cell culture

Human colonic adenocarcinoma HT-29 cells were obtained from ECACC (European Collection of Cell Cultures) and grown in RPMI-1640 (Lonza Group Ltd, Switzerland) with 10% fecal calf serum (FCS, Lonza Group Ltd, Switzerland) and 0.08% antibiotics (gentamycine sulfate, Lonza Group Ltd, Switzerland) at 37°C and 5% CO_2_.

#### Cytotoxicity of fecal water: WST-1 assay

Cytotoxicity of fecal water was measured using a colorimetric cell viability assay, the WST-1 assay, on HT-29 cells. Cells were seeded in 96-well plates (10^4^ cells per well) and grown for 1 day before start of the incubation. HT-29 cells were exposed to serial dilutions of fecal water samples (1/4–1/1024) for 72 h at 37°C and 5% CO_2_. Each analysis was performed in triplo. Medium was used as a negative control and Triton X-100 (0.5%) was used as a positive control. After washing the cells with medium, cell viability was checked by adding 100 µL of 10% solution of the tetrazolium salt, 4-[3-[4-Iodophenyl]-2-4-(4-nitrophenyl)-2H-5-tetrazolio-1,3-benzene disulfonate (WST-1, Roche Diagnostics, Switzerland), to the cells. The cells were incubated at 37°C at 5% CO_2_ in the dark. After 2 h and 4 h absorbance at 450 nm was measured with a spectrophotometer (2103 Envision Multilabel Reader, Perkin Elmer Waltham, MA). Results were expressed as fold dilution at which 50% of the cells died.

#### Genotoxicity of fecal water: comet assay

Genotoxic activity of fecal water was analyzed using alkaline single cell gel electrophoresis (Comet Assay) based on a method developed by Singh et al [34]. Experimental conditions were chosen based on previous optimization experiments (data not shown). Cells were seeded in 24-well plates (2×10^4^ cells per well) and grown for 3 days before the start of the incubation. HT-29 cells were incubated with 10% fecal water for 24 h. All samples were analyzed in duplo, medium was included as a negative control and 100 µmol/L H_2_O_2_ as a positive control for each run. After collection and centrifugation, the cells were fixed in low-melting point agarose at a concentration of 2,5×10^3^ cells/mL on pre-coated slides (Trevigen Inc., Gaithersburg, MD, USA). Two slides were prepared from each incubation well. The slides were incubated into cold lysis buffer (2,5 mol/L NaCl, 0,1 M EDTA, 0,01 mol/L Tris, 0,25 mol/L NaOH, 10% DMSO, 1% Triton X-100) for 1 h at 4°C and in alkaline buffer (0,2 mol/L NaOH, 1 mmol/L EDTA) for 20 min at RT. Next, electrophoresis was carried out for 30 min at 21 V. Finally, the slides were washed twice with H_2_O and once with 70% ethanol. All steps were carried out in the dark.

For quantification of DNA damage, the slides were stained with Sybr Green I (Trevigen Inc., Gaithersburg, MD, USA) and microscopically evaluated using dedicated software (LUCIA Comet Assay, Nikon Instruments, Melville, NY, USA). On each slide DNA damage in 50 nuclei was determined under the microscope (Nikon eclipse Ti inverted microscope, Nikon Instruments, Melvile, NY, USA). Tail length (TL), the length between the centre of the head and the end of the tail, was quantified as a measure of DNA damage.

#### Data analysis

Statistical analysis was performed using SPSS version 17.0 (SPSS Inc., Chicago, USA). Assumptions of normality and equal standard deviation were checked using the Kolgormorov-Smirnov and Levene Test, respectively. When assumptions were met differences between the NP, HP and LP diet were evaluated using repeated measures ANOVA, followed by paired T-tests when the ANOVA showed a significant result. In case of missing values, an unstructured linear mixed model was applied using the treatment as fixed effect. When assumptions of normality and equal standard deviations were not met a Friedman-test was performed, followed by a Wilcoxon-tests when the Friedman-test showed a significant result. Results were corrected for multiple testing using the Bonferoni-correction. The level of statistical significance was set at p<0.05. When assumptions of normality and equal standard deviations were met correlations were tested using the Pearson’s correlation test. Otherwise a Spearman’s rank correlation test was used. Bland-Altman plots were used to illustrate the difference between energy need measured using indirect calorimetry and energy intake calculated from the 7-day dietary journals [35]. Unscrambler version 9.7 (CAMO A/S, Trondheim, Norway) was used to perform clustering analyses on profiles of volatile organic compounds. Sample-specific VOC were omitted from the analysis of metabolite patterns as they do not exert any discriminatory power and introduce noise if implicated into the classification model [36]. Clustering of similar metabolite patterns of the samples according to cyto- or genotoxicity was performed using partial least squares-discriminant analysis (PLS-DA) with cross-validation and was presented as a score plot. The correlating loading plots, showing the metabolites, were used to identify discriminating metabolites. Significantly different VOC were identified by conducting MANOVA-statistics using Bionumerics version 6.6 (Applied Math, Sint-Martens-Latem, Belgium). Adjusted p-values were corrected using false discovery rate (FDR) correction [37]. MANOVA-statistics were also applied to analyze differences in bandclasses and resulting p-values were corrected using false discovery rate correction. The level of statistical significance was set at p<0.1.

**Figure 2:**
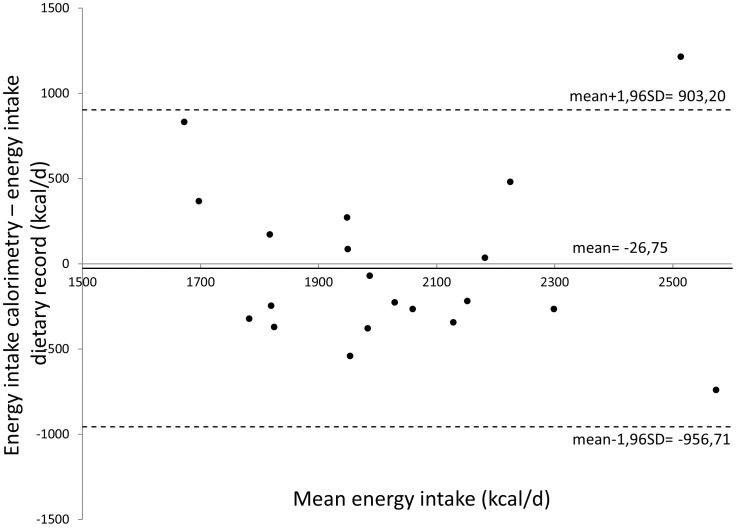
Bland-Altman plot comparing energy intake measured using indirect calorimetry (kcal/d) and energy intake calculated from the dietary records (kcal/d). Mean energy intake measured by calorimetry and dietary record are plotted against the difference between energy intake measured by calorimetry and by dietary record.

## Results

### Study Population

In total 142 subjects reacted to the advertisement and received written information about the study protocol. Twenty five of them expressed interest for participation, received oral explanation of the study protocol and were screened for eligibility. Of this group 22 subjects met the inclusion and exclusion criteria, were randomized and signed the informed consent. One subject withdrew the informed consent before the start of the study because of personal reasons and one subject dropped out after consuming the first test meal, but without collecting any samples. Twenty people completed the study according to the protocol.

Six out of 60 urine collections from four subjects were considered incomplete based on the calculation of the observed to expected creatinine ratio as proposed by Knuiman [29]. Results of these samples for urinary ^15^N-, total N-, urea- and p-cresol excretion were not included in the statistical analysis. Due to a technical problem, 2 fecal samples were lost during the homogenization of the samples. Results on fecal ^15^N-excretion of these samples were not included in the statistical analysis.

### Dietary Intake

Bland-Altman analysis showed that the caloric need of the subjects estimated from indirect calorimetry was not different from the daily energy intake during the NP diet as calculated from analysis of dietary records (Figure 2). Results of energy intake and macronutrient composition during the 3 dietary periods are summarized in Table 1. During the LP diet total dietary energy intake was significantly lower as compared to the HP diet (p = 0.021) and showed a tendency to be lower as compared to the NP diet (p = 0.051). Nevertheless, body weight remained unchanged throughout the entire study (p = 0.41). Relative protein intake during the HP diet (27% of energy intake) was significantly higher than during the NP (15%; p<0.001) and LP diet (12%; p<0.001). Protein intake was significantly different between the NP and LP diet (p = 0.003). In addition, absolute protein intake was significantly higher during the HP diet (124.4 g/d) than during the NP (74.1 g/d; p<0.001) and LP diet (50.4 g/d; p<0.001), which were also significantly different from each other (p<0.001). Total urinary N excretion, which is a measure for protein intake, was significantly different between the 3 dietary periods. The highest urinary N excretion was observed during the HP diet (14.9 g/24 h) and was significantly different from both the NP (9.5 g/24 h; p<0.001) and the LP diet (8.5 g/24 h; p<0.001). Both urinary urea and N-excretion were positively correlated with protein intake (r = 0.630; p<0.001 and r = 0.603; p<0.001, respectively).

**Table 1:** Summary of dietary records and validating measures in urine.

	Energy need (kcal/d)	NP diet	HP diet	LP diet	p-value
**Dietary records**					
Energy intake (kcal/d)	1953.7 (1859.5–2107.5)	2016.2 (1906.4–2200.1)^a^	2056.5 (1757.5–2344.9)^a^	1748.4 (1538.5–2114.1)^b^	0.043
Weight (kg)		65.6 (61.9–70.7)	65.6 (61.9–70.6)	66.2 (62.5–70.3)	0.412
Protein (%)		15.0 (13.0–17.0)^a^	27.0 (23.8–29.0)^b^	12.0 (10.8–13.0)^c^	<0.001
Protein (g/d)		74.1 (69.3–78.1)^a^	124.4 (120.4–157.9)^b^	50.4 (43.1–56.7)^c^	<0.001
Carbohydrates (%)		48.0 (46.5–52.3)^a^	41.0 (37.8–42.0)^b^	59.5 (56.0–61.0)^c^	<0.001
Carbohydrates (g/d)		246.0 (219.7–267.5)^a^	203.4 (168.2–246.0)^b^	238.5 (212.7–307.6)^a^	0.011
Fat (%)		34.5 (31.0–39.0)^a^	32.0 (28.8–34.3)^a^	28.5 (22.8–30.0)^c^	<0.001
Fat (g/d)		79.3 (63.6–88.1)^a^	72.4 (63.9–81.9)^a^	51.5 (47.9–61.7)^c^	<0.001
Fiber (g/d)		16.3 (14.8–19.1)	15.4 (11.1–19.2)	17.4 (14.7–22.9)	0.116
Calcium (mg/d)		530.5 (441.7–617.0)^a^	1038.7 (925.0–1214.8)^b^	277.8 (203.8–366.5)^c^	<0.001
**Urinary parameters**					
N (g/24 h)		9.5 (7.7–11.4)^a^	14.9 (11.8–19.7)^b^	8.5 (7.4–10.1)^a^	<0.001
Urea (g/24 h)		18.3 (14.6–22.8)^a^	31.2 (22.0–40.6)^b^	15.8 (13.5–20.9)^a^	<0.001

All values are expressed as medians (IQR) (n = 20). Parameters with different letters (a, b, c) in superscript are significantly different between the dietary interventions, test. Friedman and Wilcoxon tests were used to evaluate the results, except for urinary N and urea. Due to missing, values an unstructured linear mixed model was applied using treatment as fixed effect. P-values refer to Friedman tests.

Relative carbohydrate intake was highest during the LP diet (59%) and significantly higher than during the NP (48%; p<0.0001) and HP diet (41%; p<0.0001). Absolute carbohydrate intake was lower during the HP diet (203.4 g/d) than during the NP (246.0 g/d; p = 0.006) and the LP diet (238.5 g/d; p = 0.018). The NP and LP diet were not different. Relative fat intake was significantly lower during the LP diet (28.5%) as compared to the NP (34%; p<0.001) and HP diet (31%; p<0.001). Also absolute fat intake was significantly lower during the LP diet (51.5 g/d) than during the NP diet (79.3 g/d; p<0.001) and the HP diet (72.4 g/d; p<0.001). Consumption of fiber was constant during the dietary interventions (p = 0.12). Calcium intake was significantly higher during the HP diet (1038.7 mg/d) compared to the NP (530.5 mg/d; p<0.001) and LP diet (277.8 mg/d; p<0.001). Calcium intake was also significantly higher during the NP diet than during the LP diet (p<0.001).

### Evaluation of the Colonic Metabolism

Parameters of colonic metabolism are summarized in Table 2.

**Table 2:** Summary of the parameters of colonic metabolism and toxicity.

	NP diet	HP diet	LP diet	p-value
**Colonic metabolism**				
Fecal parameters				
*^15^N (dose %)*	10.4 (8.3–18.1)	10.6 (8.9–13.7)	13.1 (10.7–22.7)	0.059
*Total VOC*	59 (54–63)	62 (55–65)	59 (56–62)	0.284
*Total SCFA (mg/L)*	309.3 (210.5–442.5)	365.3 (256.2–555.4)	378.5 (252.6–508.5)	0.212
*Acetic acid (mg/L)*	217.8 (140.4–299.3)	251.9 (148.2–378.4)	261.3 (165.0–372.2)	0.861
*Propionic acid (mg/L)*	48.7 (38.8–79.5)	68.8 (46.6–89.0)	55. 3 (44.5–86.0)	0.522
*Butyric acid (mg/L)*	51.0 (22.5–60.6)	42.3 (29.8–71.2)	46.7 (34.8–64.0)	0.705
*Total BCFA (mg/L)*	18.8 (14.8–35.5)	29.2 (19.8–44.1)	19.8 (35.4–14.5)	0.091
*Isobutyric acid (mg/L)*	9.6 (7.0–16.6)^a^	14.7 (10.2–20.5)^b^	10.4 (7.7–18.3)^ab^	0.022
*Isovaleric acid (mg/L)*	9.2 (7.5–17.8)	12.7 (9.5–22.4)	10.2 (8.1–15.8)	0.074
*Dimethyl sulfide (µg/L)*	4.6 (3.4–6.5)	4.6 (4.3–7.7)	6.3 (4.9–14.4)	0.204
*p-Cresol (mg/L)*	1.9 (1.5–2.3)	2.3 (1.5–2.8)	1.9 (1.7–2.3)	0.246
*Total fecal output (g)*	227.6 (137.3–312.8)	254.2 (196.9–319.5)	216.4 (156.3–350.3)	0.524
*Fecal dry weight (%)*	26.9 (25.3–30.8)^a^	30.1 (28.2–33.4)^b^	29.0 (24.4–31.3)^ab^	0.017
*^3^H-recovery (%)*	65.8 (45.4–84.0)	65.2 (41.2–85.0)	58.8 (47.2–77.2)	0.985
Urinary parameters				
*^15^N (dose %)*	35.5 (30.2–41.9)	39.7 (31.7–47.2)	36.5 (29.1–43.6)	0.280
*p-Cresol (mg/24* *h)*	32.1 (25.8–37.7)^a^	45.8 (39.4–53.8)^b^	33.7 (21.5–48.9)^ab^	0.018
**Fecal water toxicity**				
Genotoxicity (TL)	99.1 (47.4–121.7)	103.1 (49.6–123.8)	109.4 (47.1–148.4)	0.861
Cytotoxicity (IC_50_)	42.1 (25.5–49.4)	27.1 (19.6–40.3)	28.9 (25.7–50.3)	0.165

All values are expressed as median (IQR) (n = 20). Parameters with different letters (a, b, c) in superscript were significantly different between the dietary interventions. Friedman and Wilcoxon tests were used to evaluate the results, except for urinary ^15^N and p-cresol, and fecal ^15^N. Due to missing values an unstructured linear mixed model was applied using treatment as fixed effect. P-values refer to Friedman tests.

#### Fecal parameters

Subjects collected all feces during the 72 h after a test meal. Total fecal output did not change between the different dietary interventions (p = 0.05). However, fecal dry weight was significantly higher during the HP diet (30.6%) than during to the NP (26.9%; p = 0.019). No difference was found in ^3^H-recovery (p = 0.99), indicating no change in transit time during the different intervention periods.

#### Ammonia metabolism: Total N and ^15^N excretion in urine and feces

To evaluate the bacterial ammonia metabolism excretion of ^15^N was measured in urine and feces. No difference was found in urinary excretion of ^15^N (p = 0.14). Fecal ^15^N excretion tended to be higher during the LP diet (13.1%) compared to the HP diet (11.0%; p = 0.07), whereas no differences were detected between the NP and LP diet (p = 0.56) or between the NP and HP diet (p = 0.25).

#### Protein fermentation: Urinary p-cresol excretion

**Figure 3:**
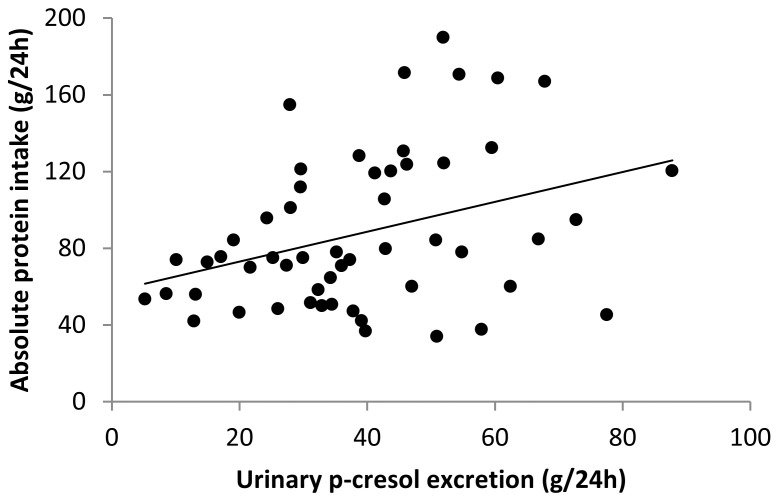
Scatter plot of the comparison between urinary p-cresol excretion (mg/24 h) and absolute protein intake (g/24 h). Urinary p-cresol excretion correlated positively with absolute protein intake (Spearman’s r = 0.371, p = 0.007).

Urinary excretion of p-cresol was measured to estimate the degree of protein fermentation [31], [1]. Urinary excretion of p-cresol was significantly higher during the HP diet (45.8 mg/24 h) than during the NP diet (32.1 mg/24 h; p<0.05) and tended to be higher than during the LP diet (33.7 mg/24 h; p = 0.089). The LP and the NP diet were not different. Urinary p-cresol excretion was significantly correlated to absolute protein intake (r = 0.371; p = 0.007) (Figure 3).

### Evaluation of Fecal Water Genotoxicity and Cytotoxicity

Fecal water genotoxicity was not different between the dietary intervention periods (p = 0.86) (Table 2). In addition, fecal water genotoxicity was not correlated to protein intake (p = 0.86) neither to protein fermentation as estimated from the urinary p-cresol excretion (p = 0.49).

**Figure 4:**
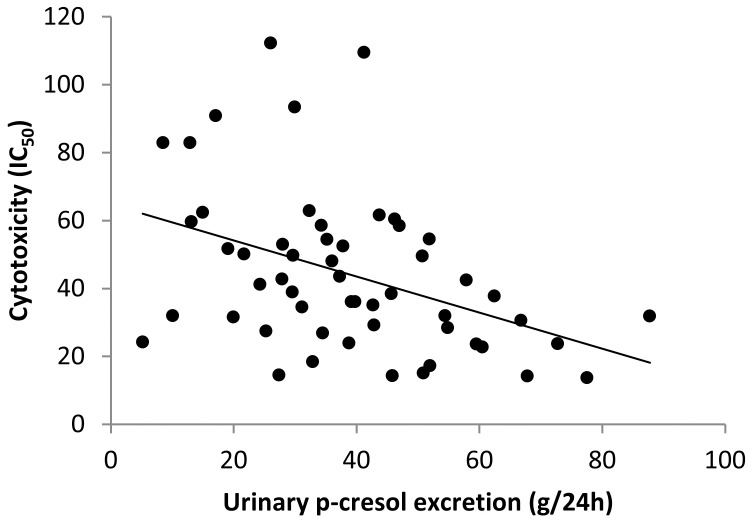
Scatter plot in which cytotoxicity (IC50) is plotted against urinary p-cresol excretion (mg/24 h). The plot shows a negative correlation between both parameters (Spearman’s r = −0.435, p = 0.001).

Similarly, fecal water cytotoxicity was not different between the dietary periods (p = 0.17). Nevertheless, fecal water cytotoxicity was negatively correlated with urinary p-cresol excretion (Spearman’s r = −0.435, p = 0.001) (Figure 4) and tended to be negatively correlated with protein intake (Spearman’s r = −0.237; p = 0.069).

### Metabolite Profiles Associated with Toxicity

#### Metabolite profiles

In total 146 different VOC were identified in the fecal samples. On average 59±6 VOC were found in each sample. Fourteen VOC were present in all samples, 57 VOC were present in at least 50% of the samples and 23 VOC were sample specific. The number of VOC per sample was not different during the different dietary periods (p = 0.28).

VOC identified in the fecal samples collected during the different dietary periods are listed in Table S1. Fecal concentrations of the SCFA, acetic acid (p = 0.86), propionic acid (p = 0.52) and butyric acid (p = 0.71) were not different between the different dietary periods (Table 2). Total fecal BCFA concentrations tended to be higher during the HP diet (29.21 mg/l) than during the NP (18.77 mg/L; p = 0.091) and the LP diet (19.8 mg/L). Fecal isobutyric acid concentration was significantly higher during the HP diet (14.66 mg/L) than during the NP diet (9.56 mg/L; p = 0.027) and fecal isovaleric acid concentration tended to be higher during the HP diet (12.86 mg/L) than during the LP diet (10.22 mg/L; p = 0.075). No difference was found in fecal dimethyl sulfide (p = 0.20) and p-cresol (p>0.25) concentration between the different dietary interventions.

#### Metabolites associated with genotoxicity

**Figure 5:**
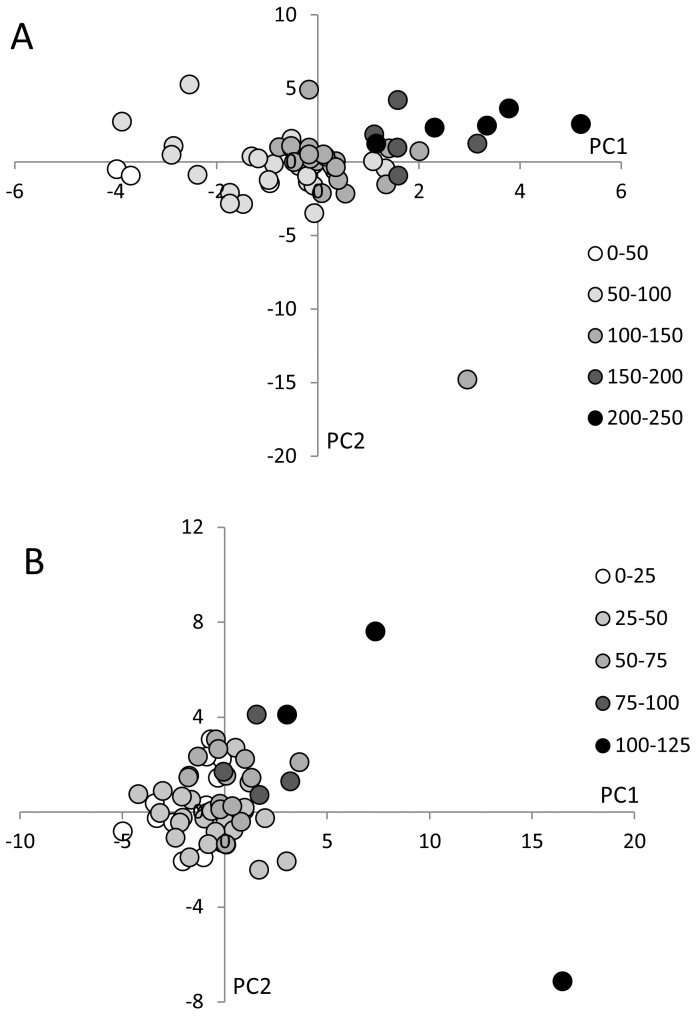
Score plots showing clustering of the metabolite profiles analyzed using PLS-DA according to genotoxicity (A) and cytotoxicity (B). (A) High genotoxicity samples are located on the right side of the score plot, while low genotoxicity samples are present on the left side, indicating a difference in VOC profile between high and low genotoxicity samples. (B) High cytotoxicity samples are present on the upper right side of the score plot and low cytotoxicity samples on the lower left side, indicating a difference in VOC profile between high and low cytotoxicity samples.

Metabolite profiles were clustered using PLS-DA-analysis with genotoxicity as category variable. To this purpose genotoxicity data were divided in 5 categories from very low to very high genotoxicity. Low genotoxicity samples clustered on the left side of the score plot whereas high genotoxicity samples were found on the right side of the plot (Figure 5A). The corresponding loading plot revealed a number of sulfides associated with highly genotoxic samples (data not shown). Subsequent MANOVA-analysis with FDR correction yielded the following metabolites that were significantly more abundant in the high toxicity samples: 3-methyl-2-butanone (p = 0.045), dimethyl disulfide (p = 0.063), allyl acetate (p = 0.048) and heptanal (p = 0.009). No metabolites were significantly more abundant in the samples with low genotoxicity.

#### Metabolites associated with cytotoxicity

PLS-DA analysis of the metabolite patterns based on cytotoxicity shows low toxicity samples on the left lower quarter of the score plot and high toxicity samples on the right upper quarter (Figure 5B). This discrimination was associated with the presence of acids and alcohols in the highly cytotoxic samples and the presence of cycloalkanes and cycloalkenes in the low cytotoxic samples (data not shown). MANOVA-analysis retrieved 13 metabolites significantly associated with high cytotoxicity. These compounds are indole (p = 0.015), 1-octanol (p = 0.007), heptanal (p = 0.054), 2,4-dithiapentane (p<0.001), allyl-isothiocyanate (p = 0.007), 1-methyl-4-(1-methylethenyl)-benzene (p = 0.050), propionic acid (p = 0.026), octanoic acid (p = 0.012), nonanoic acid (p = 0.007) and decanoic acid (p = 0.007). No metabolites were significantly more abundant in the samples with low cytotoxicity.

#### Effect of the intervention on fecal microbiota

In total 64 bandclasses were allocated. No bandclasses were differentially present in the samples collected during the different dietary periods, indicating no effect of the intervention on the composition of the predominant bacteria in the gut.

## Discussion

Lifestyle and diet are important contributors to the etiology of gut diseases, such as CRC and IBD. Colonic bacterial fermentation results in the production of a wide variety of metabolites that intimitately interact with the host’s epithelial cells and in this way affect health. Whereas it is widely accepted that carbohydrate fermentation results in beneficial effects for the host because of the generation of SCFA, protein fermentation is considered detrimental for the host's health. However, the relationship between protein fermentation and gut health has not been thoroughly investigated [38]. In the present intervention trial that specifically modified protein fermentation we could not establish an association between degree of protein fermentation and markers of gut toxicity.

Protein intake was estimated from 7-d weighed food records, reported as golden standard [39] and validated by urinary biomarkers (urea and total nitrogen). Although several studies report underestimation of protein intake when based on assessment of dietary records [40], we obtained excellent correlation between dietary records and urinary markers. In addition, the agreement between the measured caloric use and the calculated energy intake from the dietary journals suggest that the dietary assessment was reliable. Besides, the dietary intervention successfully modified the degree of protein fermentation as evidenced by the significant positive association between protein intake and urinary p-cresol excretion. In this study, diets were specifically drawn up to modify protein intake and protein fermentation without affecting fat intake and fiber intake. Fat is considered as a potential factor that increases CRC risk due to increased production of secondary bile acids [41], [42] whereas fiber is considered as a protective factor through the production of SCFA [43]. The HP diet protein mainly consisted of lean meat, chicken and dairy products (which explains the significantly higher calcium intake during this period). Fat and fiber intake remained similar as compared to the NP diet. The fact that fecal SCFA concentrations remained constant during the intervention confirms that carbohydrate fermentation was not affected. In the LP diet, protein was mainly replaced by digestible carbohydrates to keep the fiber intake constant. Unfortunately, subjects also reduced fat intake.

The labeled biomarker lactose-[^15^N,^ 15^N]-ureide was added to the test meal to evaluate changes in the bacterial ammonia metabolism in the colon due to the intervention. This biomarker is used as a vector to administer a known amount of ^15^NH_3_ in the colon. Labeled ammonia can be used by the colonic microbiota for their growth and activity and is consequently retrieved in feces. Ammonia that is not taken up by the bacteria is absorbed through the colonic mucosa, converted in the liver to ^15^N-urea and excreted in urine [44], [45]. Excretion of ^15^N in urine and feces was not different throughout the study suggesting that the changes in protein intake and protein fermentation did not affect colonic bacterial activity. To detect changes in predominant composition of the microbiota, we applied DGGE-analysis on bacterial DNA extracted from fecal samples. An *in vitro* study and animal studies indicate that higher protein or red meat intake shifts the balance of the gut microbiota away from potentially beneficial or health promoting bacteria such as lactobacilli and bifidobacteria towards a predominance of bacteria like clostridia and Bacteroides sp [46]–[48]. However, in our study no bandclasses were found to be different during the different dietary interventions, indicating no effect of the intervention on the composition of the microbiota in the gut.

In this study, protein intake and protein fermentation were related to genotoxicity and cytotoxicity towards colonic cells. Genotoxic compounds induce genetic damage directly or indirectly by various mechanisms. Compounds which are positive in tests that detect such kinds of damage have the potential to be human carcinogens. Genotoxicity tests have therefore mainly been used to predict carcinogenicity. Cytotoxicity tests measure the potential of compounds to induce cell death. The WST-test used in this study measures the reducing potential of the cell using a colorimetric reaction. Alternative assays are based on assessment of the cell membrane integrity.

Studies in rats previously showed that increased protein intake and increased protein fermentation were associated with genotoxicity [2], [49]. Studies in humans are scarce. A recent human study compared fecal water genotoxicity after a high protein weight loss diet (35% protein) and a high carbohydrate weight loss diet (17% protein) [50]. Although a weak correlation was found between urinary p-cresol excretion and DNA damage, the reduction in DNA damage was similar in both groups and was associated to loss of weight irrespective of the diet. It therefore seems that weight loss contributes more to a reduction in fecal water genotoxicity than protein fermentation. In the present human study, protein intake and protein fermentation were not associated with genotoxicity. However, subjects consumed an isocaloric diet throughout the study period which might explain the different results.

**Figure 6:**
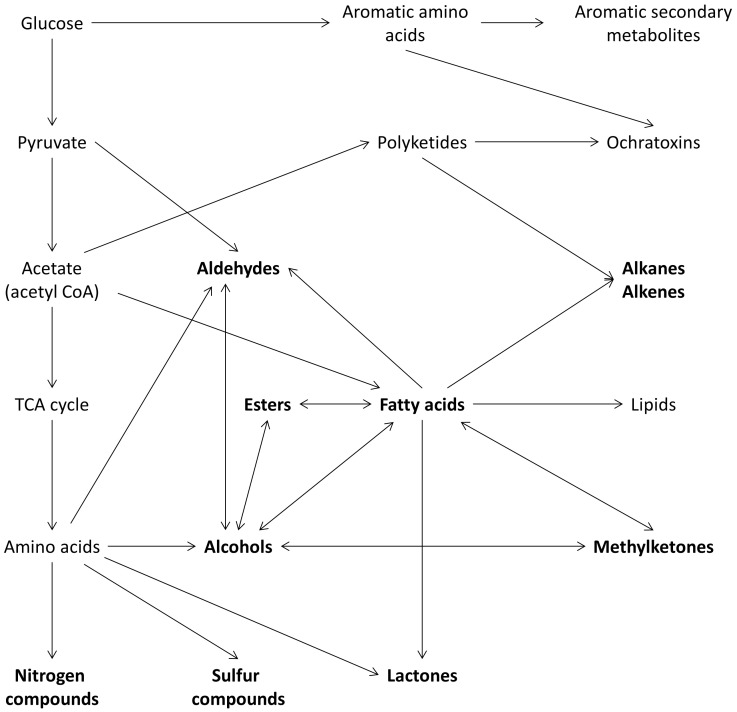
Main metabolic pathways for the production of microbial metabolites. VOC are shown in bold [52].

Whereas genotoxicity was not correlated to protein fermentation, cytotoxicity of fecal water, was reduced with increased protein fermentation. Similar results were obtained by Glinghammer *et al.* who compared fecal water genotoxicity and cytotoxicity of a dairy product-rich and a dairy product-free diet. Genotoxicity was not different between both diets, whereas cytotoxicity of the samples from the dairy-product rich diet was reduced (p = 0.025). This reduction in cytotoxicity was attributed to calcium present in the diet [51]. The diary product-rich diet also resulted in a significantly higher protein intake compared to the dairy product-free diet (91 g/d versus 60 g/d; p<0.001). Unfortunately no parameters of protein fermentation were evaluated. As the protein intake during the HP diet was partially achieved by increasing dairy product, calcium intake was significantly higher as compared to the NP and LP diet. Therefore it is likely that the lower cytotoxicity observed should be attributed to a higher calcium intake rather than to an increased protein intake. The positive effect of calcium on cytotoxicity was directly assessed in a 12-week oral calcium-supplementation study were fecal water cytotoxicity was also reduced [52]. This effect was explained by the precipitation of bile acids and fatty acids. We analyzed fecal metabolite profiles to clarify the relation between intestinal bacterial metabolites and parameters of gut health. Identified VOC were alcohols, ketones, terpenes, esters, hydrocarbons, aldehydes, sulfur- and nitrogen compounds. The complex metabolic pathways for microbial VOC formation are depicted in Figure 6. Most information on microbial VOC arises from environmental studies that used those compounds as indicators of biocontamination. Since the 1990s, microbial VOC have been analyzed in indoor air to detect hidden microbial growth and have been related to health risks such as eye and upper respiratory tract irritation [53].

By clustering metabolite profiles according to genotoxicity or cytotoxicity we identified a number of metabolites that were significantly associated with genotoxicity or cytotoxicity. As surprisingly little is known about VOC produced by intestinal microbiota, it is extremely difficult to assign individual components to specific bacteria and to estimate the relevance to gut health of individual colonic compounds. In addition, many compounds also have other environmental sources than microbial metabolism (e.g. diet composition) and do not only originate from bacteria [53]. In a recent study in mice, 179 metabolites were identified in the colonic luminal metabolome of which 131 were common to those detected in the chow of the animals [54]. Nevertheless, a number of sulfides, dimethyl sulfide, dimethyl disulfide, dimethyl trisulfide and methyl propyl disulfide, appeared to be associated with genotoxicity in our study. This was confirmed in the MANOVA-analysis, which identified higher concentrations of dimethyl disulfide in the samples with high genotoxicity. Sulfides are produced from fermentation of the sulfur-containing amino acids, cysteine and methionine, but also originate from mucines or food-derived sulfate [55]. Although it was previously shown that dietary protein from meat is an important substrate for sulfide generation in the colon [56], our results suggest a considerable contribution from other sources of sulfur as well. For example western diets have been reported to be high in sulfate [55]. Also allyl acetate was positively associated with genotoxicity. Allyl acetate is a precursor of the known toxic compound acrolein. In rats a daily dose of 100 mg allyl acetate/kg caused hemorrhage, inflammation and epithelial necrosis in the large and small intestine [57]. Heptanal, 1-octanol and the medium chain fatty acids most likely originate from fatty acid metabolism. Their association with genotoxicity and cytotoxicity could be explained by their generation during lipid peroxidation. Possibly, they rather result from toxicity instead of causing toxicity. Surprisingly, also the SCFA, propionic acid, was associated to cytotoxicity. In vitro cytotoxicity studies in different cell lines showed that propionic acid induces cytotoxicity through a mechanism of apoptosis [58].

In conclusion, the results obtained in this study do not provide evidence for a role of protein fermentation in gut toxicity in healthy human subjects. On the other hand, we were able to identify several metabolites that possibly play a role in colonic genotoxicity and cytotoxicity. However, more research is needed on the actual source of these metabolites.

## Supporting Information

Table S1 **Percentage occurence and mean relative indices (I) of VOCs in fecal samples.**
(DOC)

Checklist S1 **CONSORT checklist.**
(DOC)

Protocol S1 **Study protocol.**
(DOC)
