# Supplementary material for: Modulation of Protein Fermentation Does Not Affect Fecal Water Toxicity: A Randomized Cross-Over Study in Healthy Subjects
Source: PLoS One. 2012 Dec 20;7(12):e52387. doi: 10.1371/journal.pone.0052387 (PMC3527498; doi:10.1371/journal.pone.0052387)
Supplement: Table S1 — Percentage occurence and mean relative indices (I) of VOCs in fecal samples. (DOC) [file pone.0052387.s001.doc]

Table S1: Percentage occurence and mean relative indices (I) of VOCs in fecal samples

| **VOC** | **NP** | | **HP** | | **LP** | |
| --- | --- | --- | --- | --- | --- | --- |
|  | **I (x100)** | **%** | **I (x100)** | **%** | **I (x100)** | **%** |
| 1,3,8-p-menthatriene | 0 | 0 | 0,012 | 15 | 0,006 | 5 |
| 1,3-pentadiene | 2,060 | 90 | 1,374 | 75 | 1,955 | 80 |
| 1-butanol | 24,356 | 100 | 22,976 | 100 | 39,647 | 100 |
| 2-methyl-1-butanol | 0,034 | 10 | 0,011 | 10 | 0,012 | 10 |
| 2,3-dimethyl-1-butene | 0,027 | 10 | 0,049 | 10 | 0,040 | 10 |
| 1-heptanol | 0,032 | 35 | 0,048 | 15 | 0,013 | 10 |
| 1-hexanol | 0,2649 | 25 | 0,245 | 45 | 0,092 | 40 |
| 2-ethyl-1-hexanol | 0,098 | 85 | 0,090 | 70 | 0,123 | 90 |
| 3-methyl-1H-indole | 1,647 | 65 | 1,586 | 75 | 0,662 | 60 |
| 1-octanol | 0 | 0 | 0,716 | 15 | 0 | 0 |
| 1-pentanol | 0,027 | 5 | 0,048 | 10 | 0,094 | 15 |
| 1-propanol | 0,648 | 15 | 0,634 | 25 | 0,863 | 30 |
| 2-methyl-1-propanol | 0,078 | 50 | 0,119 | 60 | 0,185 | 50 |
| 2,3-butanedione | 0,096 | 40 | 0,196 | 35 | 0,113 | 40 |
| 2,3-pentanedione | 0 | 0 | 0 | 0 | 0,007 | 10 |
| 2,4-dithiapentane | 0,011 | 5 | 0,004 | 5 | 0,004 | 5 |
| 2-acetyl-5-methylfuran | 0 | 0 | 0 | 0 | 0,009 | 10 |
| 2-butanone | 20,765 | 85 | 37,435 | 95 | 32,413 | 90 |
| 3,3-dimethyl-2-butanone | 0,248 | 10 | 0,704 | 5 | 0,295 | 5 |
| 3-methyl-2-butanone | 0,173 | 5 | 0,589 | 5 | 0 | 0 |
| 2-butenal | 0,012 | 5 | 0,008 | 5 | 0 | 0 |
| 5-methyl-2-Furancarboxaldehyde | 0,131 | 75 | 0,109 | 80 | 0,092 | 70 |
| 2-hexanone | 0,064 | 35 | 0,099 | 30 | 0,083 | 30 |
| 2-pentanone | 4,397 | 45 | 2,273 | 60 | 3,516 | 55 |
| 3-methyl-2-pentanone | 0 | 0 | 0,043 | 5 | 0 | 0 |
| **VOC** | **NP** | | **HP** | | **LP** | |
|  | **I (x100)** | **%** | **I (x100)** | **%** | **I (x100)** | **%** |
| 3,4-dimethylthiophene | 0,761 | 90 | 0,516 | 90 | 0,583 | 95 |
| 3-carene | 0,129 | 40 | 0,491 | 60 | 0,877 | 40 |
| 3-furaldehyde | 0,120 | 80 | 0,191 | 85 | 0,146 | 75 |
| 3-pentanone | 0,263 | 20 | 0,408 | 30 | 0,382 | 25 |
| 6-methyl-5-hepten-2-one | 0,029 | 5 | 0 | 0 | 0 | 0 |
| Acetaldehyde | 114,796 | 100 | 121,641 | 90 | 136,880 | 100 |
| Acetic acid | 8,743 | 100 | 9,876 | 100 | 11,127 | 100 |
| Acetic acid, ethenyl ester | 0 | 0 | 0 | 0 | 0,028 | 5 |
| Acetic acid, ethyl ester | 0 | 0 | 0 | 0 | 0,086 | 5 |
| Acetic acid, 2-propenyl ester | 0 | 0 | 0,004 | 5 | 0,018 | 5 |
| Acetic acid, methyl ester | 0 | 0 | 0 | 0 | 0,0003 | 5 |
| Acetone | 106,722 | 100 | 76,135 | 100 | 106,655 | 100 |
| Acetonitrile | 33,106 | 65 | 44,023 | 95 | 29,699 | 75 |
| Acetophenone | 1,129 | 75 | 1,530 | 75 | 2,449 | 70 |
| Alcohol RT 7,52 | 0 | 0 | 0 | 0 | 0,118 | 10 |
| Alcohol RT 9,20 | 0,459 | 20 | 0 | 0 | 0,005 | 10 |
| Alkeen RT 20.33 | 0 | 0 | 0,012 | 5 | 0 | 0 |
| Allyl Isothiocyanate | 0,107 | 20 | 0,013 | 10 | 0 | 0 |
| alpha-Phellandrene | 0,468 | 90 | 1,116 | 80 | 0,542 | 80 |
| alpha-Pinene | 0,553 | 25 | 3,100 | 50 | 1,832 | 35 |
| p-allyl-anisole | 0 | 0 | 0,046 | 5 | 0 | 0 |
| Benzaldehyde | 60,707 | 100 | 89,376 | 100 | 96,449 | 100 |
| 3,5-dimethyl-benzaldehyde | 0,004 | 10 | 0 | 0 | 0 | 0 |
| Benzene | 0,028 | 15 | 0,077 | 25 | 0,0160 | 15 |
| (2-methyl-2-propenyl)-benzene | 0 | 0 | 0,006 | 5 | 0 | 0 |
| 1,2,3-trimethyl-benzene | 0,086 | 50 | 0,047 | 35 | 0,126 | 55 |
| 1-ethyl-2-methyl-benzene | 1,719 | 95 | 2,004 | 100 | 2,076 | 95 |
| 1-methyl-2-(1-methylethyl)-benzene | 6,129 | 100 | 11,158 | 100 | 8,877 | 95 |
| **VOC** | **NP** | | **HP** | | **LP** | |
|  | **I (x100)** | **%** | **I (x100)** | **%** | **I (x100)** | **%** |
| 1-methyl-4-(1-methylethenyl)-benzene | 0,063 | 10 | 0,012 | 15 | 0,026 | 10 |
| 4-ethenyl-1,2-dimethyl-benzene | 0 | 0 | 0 | 0 | 0,023 | 5 |
| Benzene-like RT 11.25 | 0 | 0 | 0 | 0 | 0,011 | 5 |
| 2-methyl-benzofuran | 0,015 | 10 | 0,071 | 20 | 0,016 | 20 |
| Benzonitrile | 0,0002 | 5 | 0,002 | 5 | 0 | 0 |
| Benzyl Alcohol | 0,293 | 5 | 0 | 0 | 0 | 0 |
| Betazole | 0 | 0 | 0,032 | 5 | 0 | 0 |
| Bromochloronitromethane | 0,220 | 100 | 0,214 | 100 | 0,257 | 100 |
| Butanal | 8,550 | 35 | 11,572 | 30 | 17,775 | 45 |
| 3-methyl-butanal | 2,757 | 15 | 2,064 | 20 | 4,055 | 10 |
| Butanoic acid | 76,541 | 100 | 89,828 | 100 | 85,426 | 100 |
| 2-methyl-butanoic acid | 98,666 | 100 | 125,389 | 95 | 96,531 | 100 |
| 3-methyl-butanoic acid | 96,616 | 100 | 121,645 | 100 | 83,203 | 100 |
| Butanoic acid, ethyl ester | 0,007 | 5 | 0,033 | 15 | 0,030 | 10 |
| Butylcaprylate | 0 | 0 | 0,229 | 5 | 0 | 0 |
| Camphene | 0 | 0 | 1,106 | 20 | 0,734 | 15 |
| Carbon disulfide RT 2,38 | 0,708 | 50 | 1,682 | 60 | 2,144 | 75 |
| Cinnamaldehyde | 0,001 | 5 | 0 | 0 | 0 | 0 |
| Cycloalkeen RT 10,74 | 0,206 | 45 | 0,983 | 55 | 0,216 | 40 |
| Cycloalkeen RT 18.16 | 0 | 0 | 0 | 0 | 0,014 | 5 |
| Cycloalkeen RT 18.48 | 1,145 | 60 | 1,365 | 65 | 0,845 | 40 |
| Cycloalkeen RT 19,33 | 0,144 | 60 | 0,143 | 60 | 0,177 | 55 |
| Cycloalkeen RT 19,98 | 0,339 | 70 | 1,097 | 70 | 1,718 | 65 |
| Cycloalkeen RT 19.06 | 0 | 0 | 0 | 0 | 0,053 | 5 |
| Cycloalkeen RT 6.73 | 0,005 | 5 | 0,056 | 15 | 0,018 | 10 |
| Cyclohexane | 14,410 | 65 | 12,685 | 65 | 11,053 | 55 |
| 2,3-dimethyl-cyclohexanol | 0,873 | 15 | 1,419 | 30 | 0,753 | 25 |
| **VOC** | **NP** | | **HP** | | **LP** | |
|  | **I (x100)** | **%** | **I (x100)** | **%** | **I (x100)** | **%** |
| 5-methyl-2-(1-methylethyl)-, (1.alpha.,2.beta.,5.alpha.)-(.+/-.)-cyclohexanol | 0,069 | 5 | 0,049 | 5 | 0,548 | 10 |
| Cyclopentanone | 0,002 | 5 | 0,013 | 20 | 0,002 | 5 |
| Decanoic acid | 0,098 | 10 | 7,394 | 10 | 0,136 | 15 |
| Dimethyl ether | 2,055 | 30 | 9,255 | 65 | 7,751 | 45 |
| Dimethyl sulfide | 4,423 | 75 | 4,081 | 65 | 16,119 | 90 |
| Dimethyl trisulfide | 18,942 | 100 | 19,647 | 100 | 26,868 | 100 |
| Dimethyldisulfide, dimethyl | 58,559 | 100 | 74,145 | 100 | 80,620 | 100 |
| 1-methyl-2-propenyl-disulfide | 0,030 | 5 | 0,064 | 20 | 0,043 | 15 |
| Methyl-propyl-disulfide | 0,283 | 55 | 0,309 | 70 | 0,349 | 75 |
| DL-2,3-Butanediol | 0 | 0 | 0 | 0 | 0,683 | 5 |
| Dodecanal | 0,423 | 5 | 0,040 | 5 | 0 | 0 |
| Ethanethiol | 0 | 0 | 0 | 0 | 0,002 | 5 |
| Ethanol | 0 | 0 | 0,044 | 5 | 0 | 0 |
| Ethyl ether | 34,949 | 75 | 33,150 | 75 | 42,227 | 80 |
| Ethylbenzene | 0,013 | 10 | 0,008 | 10 | 0,006 | 5 |
| Furan | 4,329 | 95 | 3,427 | 90 | 3,901 | 90 |
| 2,5-dimethyl-furan | 2,495 | 95 | 2,424 | 70 | 1,193 | 65 |
| 2-ethyl-5-methyl-furan | 0,115 | 25 | 0,087 | 10 | 0,084 | 15 |
| 2-methyl-furan | 0,132 | 25 | 0,032 | 10 | 0,078 | 20 |
| 2-pentyl-furan | 0,030 | 15 | 0,068 | 30 | 0,041 | 25 |
| 3-methyl-furan | 4,777 | 100 | 3,379 | 95 | 4,191 | 100 |
| Tetrahydro-furan | 23,414 | 90 | 32,318 | 85 | 30,914 | 95 |
| Furfural | 0,012 | 20 | 0,015 | 15 | 0,003 | 5 |
| Heptanal | 0 | 0 | 0,031 | 5 | 0,018 | 5 |
| Heptanoic acid | 21,275 | 90 | 11,467 | 85 | 5,858 | 95 |
| Hexanal | 0,197 | 40 | 0,110 | 25 | 0,202 | 25 |
| 3-methyl-hexanal | 0,032 | 10 | 0,004 | 5 | 0,007 | 5 |
| **VOC** | **NP** | | **HP** | | **LP** | |
|  | **I (x100)** | **%** | **I (x100)** | **%** | **I (x100)** | **%** |
| Hexanoic acid | 78,000 | 100 | 57,158 | 100 | 30,798 | 100 |
| Hexanoic acid branched | 0,127 | 50 | 0,117 | 65 | 0,043 | 55 |
| Hydrogen sulfide | 0,305 | 40 | 0,579 | 55 | 0,499 | 40 |
| Indole | 0 | 0 | 0,003 | 15 | 0 | 0 |
| Limonene | 0,074 | 40 | 0,827 | 65 | 0,303 | 40 |
| Isothiocyanatomethane | 0,028 | 5 | 0 | 0 | 0 | 0 |
| Tribromomethane | 0,581 | 95 | 0,554 | 95 | 0,683 | 100 |
| Methanethiol | 3,627 | 80 | 5,721 | 65 | 5,556 | 85 |
| Methyl Alcohol | 1,929 | 25 | 1,169 | 30 | 2,574 | 35 |
| Methyl propyl ether | 0,012 | 5 | 0,063 | 10 | 0,359 | 5 |
| Methyl vinyl ketone | 0 | 0 | 0,003 | 5 | 0 | 0 |
| Methylene Chloride | 7,339 | 70 | 3,573 | 70 | 4,346 | 70 |
| Nonanoic acid | 0,009 | 25 | 0,093 | 10 | 0,003 | 5 |
| Octanoic Acid | 3,674 | 75 | 40,087 | 95 | 7,697 | 75 |
| Octanoic acid, ethylester | 0 | 0 | 0,106 | 5 | 0 | 0 |
| o-Isopropenyltoluene | 0,233 | 75 | 1,399 | 95 | 1,157 | 85 |
| 2,7-dimethyl-oxepine, 2,7-dimethyl- | 0,063 | 55 | 0,059 | 45 | 0,056 | 45 |
| o-Xylene | 0,019 | 15 | 0,0133 | 20 | 0,0188 | 25 |
| Pentanoic acid | 47,941 | 100 | 49,561 | 100 | 41,131 | 100 |
| 4-methyl-pentanoic acid | 1,252 | 100 | 1,089 | 100 | 0,684 | 100 |
| Pentanoic acid, butyl ester | 0 | 0 | 0,009 | 5 | 0 | 0 |
| Pentanoic acid, methyl ester | 0,008 | 5 | 0,013 | 5 | 0,024 | 5 |
| Phenol | 0,024 | 45 | 0,144 | 60 | 0,116 | 45 |
| 4-ethyl-phenol | 0,046 | 20 | 0,043 | 30 | 0 | 0 |
| 4-methyl-phenol | 19,212 | 100 | 23,082 | 100 | 17,896 | 95 |
| Propanal | 29,063 | 100 | 35,016 | 95 | 38,890 | 100 |
| 2,2-dimethyl-propanal | 0,298 | 5 | 0 | 0 | 0 | 0 |
| 2-methyl-propanal | 21,809 | 100 | 26,318 | 95 | 24,102 | 95 |
| **VOC** | **NP** | | **HP** | | **LP** | |
|  | **I (x100)** | **%** | **I (x100)** | **%** | **I (x100)** | **%** |
| Propanoic acid | 12,620 | 100 | 13,849 | 100 | 15,590 | 100 |
| 2-methyl-propanoic acid | 23,035 | 100 | 32,416 | 100 | 23,409 | 100 |
| Pyrrole | 0,003 | 5 | 0 | 0 | 0,001 | 5 |
| S-Methyl pentanethioate | 0 | 0 | 0 | 0 | 0,107 | 10 |
| Styrene | 0,132 | 40 | 0,110 | 20 | 0,485 | 45 |
| Thiocyanic acid, methyl ester | 0,142 | 90 | 0,133 | 80 | 0,160 | 90 |
| 2,3-dimethyl-thiophene | 0,001 | 10 | 0,036 | 10 | 0,004 | 20 |
| 3-methyl-thiophene | 0,278 | 80 | 0,206 | 80 | 0,241 | 85 |
| Toluene | 6,964 | 95 | 4,163 | 85 | 6,907 | 85 |
| Trichloromethane | 22,096 | 70 | 20,937 | 50 | 7,365 | 60 |
| VOC RT 9.11 | 0,009 | 5 | 0 | 0 | 0,011 | 5 |
|  |  |  |  |  |  |  |
