# Supplementary material for: Modulation of Protein Fermentation Does Not Affect Fecal Water Toxicity: A Randomized Cross-Over Study in Healthy Subjects
Source: PLoS One. 2012 Dec 20;7(12):e52387. doi: 10.1371/journal.pone.0052387 (PMC3527498; doi:10.1371/journal.pone.0052387)
Supplement: Protocol S1 — Study protocol. (DOC) [file pone.0052387.s003.doc]

**Research project: Influence of a protein diet on the colonic metabolism and phosphor metabolism in healthy volunteers**

# Introduction

Recently, there is an increased interest in the use of high-protein/low-carbohydrate diets (protein diets) in the area of weight loss and treatment of disease. Protein intake results in increased satiety compared to other nutrients. Protein diets result in increased loss of fat in comparison with diets high in carbohydrates or low in proteins. Protein diets were also shown to reduce the risk on cardiovascular diseases. Positive effects were also found on body composition, lipid composition in the blood, glucose homeostasis or vitamin status in the body.

An increased protein intake probably results in an increased amount of protein in the colon. Components from endogenous or exogenous origin entering the colonic lumen will undergo bacterial metabolisation or fermentation. The resulting metabolites play an important role in energy metabolism, physiology and pathophysiology of the colon. The quality and quantity of the bacterial metabolites depends on the characteristics of the bacterial flora, the transit time through the colon and substrate availability. Carbohydrates are fermented to short chain fatty acids (SCFA: acetate, propionate, butyrate), which are generally considered to be beneficial for the colonocytes of the host. Fermentation of proteins also results in the production of SCFA, but also of potentially toxic metabolites such as amines, ammonia, mercaptanes and phenol derivatives. Ammonia and phenol derivatives are often used as cancer risk markers. Until now, the effects of a protein diet on the colon has not been investigated, therefore the goal of this present is to investigate the influence of a high dose of proteins on colon metabolism.

In both patients with chronic kidney disease and the general population serological phosphor concentration is a predictor of cardiovascular morbidity and mortality. Serological phosphor also has an important influence on the bone metabolism, also in patients with a normal kidney function. Serological phosphor concentration reflects the balance between gastro intestinal absorption, transport from and to the bone and urinary excretion. Average daily phosphor intake is 1600 mg. Dietary proteins are an important source of exogenous phosphor. Bio availability and the contribution of additives (up to 1000 mg/d) have to be considered. Parathyroid hormone (PTH) and FGF-23 induce urinary phosphor loss and, therefore, play an important role in renal phosphor homeostasis. Serum levels of PTH, but not FGF-23, increase quickly after a single oral phosphor bolus. Data concerning the physiological response to a longer term high protein diet (phosphor) are scarce.

In the proposed study protocol the influence of a high versus a low protein diet on the colonic metabolism will be investigated. As secondary end point the influence of a high versus a low protein diet on phosphor homeostasis will be studied. In Belgium, average daily protein intake is 16% of total dietary intake (<http://www.iph.fgov.be/epidemio/epinl/index5.htm>), which is higher than the recommendations by High Health counsel for Belgium. These recommendations state that the average protein need is 9-11% of total energy intake ([https://portal.health.fgov.be/pls/portal/docs/PAGE/INTERNET_PG/HOMEPAGE_MENU/ABOUTUS1_MENU/INSTITUTIONSAPPARANTEES1_MENU/HOGEGEZONDHEIDSRAAD1_MENU/BROCHURES1_DOCS/HGR%207145-2%20070118_NL.PDF](https://portal.health.fgov.be/pls/portal/docs/PAGE/INTERNET_PG/HOMEPAGE_MENU/ABOUTUS1_MENU/INSTITUTIONSAPPARANTEES1_MENU/HOGEGEZONDHEIDSRAAD1_MENU/BROCHURES1_DOCS/HGR 7145-2 070118_NL.PDF)). Energy intake will be the same during this study, while protein intake during 2 intervention periods will be increased to 30% of total daily energy intake (high protein diet) or decreased to 9% of total daily energy intake (low protein diet).

# GOALS

In the proposed project the influence of an isocaloric diet with high/low protein intake versus a normal diet on the colonic metabolism in healthy volunteers will be investigated. To this purpose parameters of colonic metabolism will be investigated:

- Analysis of ammonia metabolism in the colon after administration of lactose-[^15^N, ^15^N]-ureid
- Analysis of urinary p-cresol excretion as a marker of protein fermentation
- Analysis volatile organic compounds (VOC) in feces
- Analysis of genotoxicity of fecal water using the Comet Assay
- Analysis of cytotoxicity of fecal water using the WST-1 assay

Secondly, the influence of an isocaloric diet with high/low protein intake versus a normal diet on the phosphor homeostasis in healthy volunteers will be evaluated. To this purpose the following parameters are investigated:

- Analysis of biochemical parameters of phosphor homeostasis (serum calcium and phosphor, calcidiol, calcitriol, PTH, FGF-23)
- Analysis of parameters of bone formation and bone resorption
- Analysis of dietary phosphor and calcium
- Analysis of urinary excretion of calcium and phosphor

# METHODS

## Indirect calorimetry

Baseline energy use is measured using indirect calorimetry. Calorimetry will be performed under standard conditions (thermoneutral environment, sober and at rest). Using a ventilated-hood system the amount of O_2_ and CO_2_ in inhaled and exhaled air is measured each minute. In total there will be measured for 25 minutes. In combination with an estimation of physical activity, determined using the validated Baecke questionnaire, total energy need will be calculated. This result will be used to design isocaloric high and low protein diets.

## Body composition

Measurement of the body composition (fat mass and fat free mass) will be done using the bio-electrical tetrapolar impedance method (Bodystat 1500). A small current (800 µA, 50 kHz) is used to measure the resistance of the body. Using population specific regression equations fat mass and fat free mass are calculated. This measurement is non invasive, takes only a few minutes, is very reproducible and validated (to the deuterium dilution method).

## Analysis of the colonic ammonia metabolism after administration of lactose-[^15^N, ^15^N]-ureid

Using lactose-[^15^N, ^15^N]-ureid a known amount of [^15^N] is transported to the colon. Lactose-[^15^N, ^15^N]-ureid is not metabolized in the small bowel and reaches the colon, unchanged. In the colon the bound between lactose and [^15^N, ^15^N]-urea is specifically hydrolyzed by bacterial enzymes. The resulting [^15^N, ^15^N]-urea will mix with the natural present urea and will become available for the microbiota in the colon. [^15^N, ^15^N]-urea will be further hydrolyzed to [^15^N]-NH_3_, which can be absorbed by the bacteria and excreted in feces. [^15^N]-NH_3_ can also be absorbed by the colonocytes and, after metabolisation in the liver, urinary excreted as [^15^N]-urea. By determining how much ^15^N appears in urine and feces, the metabolism of nitrogen containing compounds can be studied. Tritium-labeled polyethyleenglycol (PEG) is administrated together with the ^15^N-biomarker to correct ^15^N-excretion for changes in transit time. [^3^H]-PEG will not be absorbed or metabolized in the gastrointestinal system and leaves the body unchanged in the feces. The recovered amount of [^3^H]-PEG can be quantified using liquid scintillation.

## Analysis of urinary p-cresol excretion as a marker of protein fermentation

Proteins escaping digestion in the small bowel and endogenous proteins and peptides produced by the microbiota are fermented in the colon to a variety of metabolites, such as p-cresol. p-Cresol is a unique bacterial metabolite (it is not produced by human enzymes) of tyrosine. p-Cresol is absorbed by the colonic mucosa, conjugated with sulphate or glucuronide and urinary excreted. Therefore, urinary p-cresol excretion is a measure for the amount of protein degradation in the colon. After extraction from the matrix, p-cresol will be measured using GC-MS.

## Analysis of VOC profiles in feces

The analysis of VOC profiles in fecal samples will give additional information on the metabolic activity of microbiota. Special attention will be given to SCFA (acetate, propionate, butyrate, …) branched chain fatty acids (Isobutyrate, isovalerate, …), sulphur containing compounds (dimethyl sulphide, trimethyl sulphide, …) and phenolic compounds (indol, skatol, phenol, p-cresol).

## Analysis of fecal water genotoxicity using the Comet Assay

The Comet Assay (or Single Cell Gel Electrophoresis Assay) is a technique that allows to detect and quantify single and double strand DNA-breaks on cellular level. To determine the DNA damage induced by fecal water (supernatans generated after ultracentrifugation of fecal samples), HT-29 clone 19A cells (a human colon cell line) will be incubated with fecal water. Next the incubated cells are cultured and put on a gel. During electrophoresis the DNA is attracted to the cathode. In case of breaks in the DNA, the DNA will move more easily through the gel. After staining a comet-like structure will appear. The length of the tail indicates the amount of DNA damage (numbers of breaks in the DNA).

# Analysis of fecal water cytotoxicity using the WST-1 assay

The WST-1 cytotoxicity test measures the metabolic capacity of living cells to reduce the yellow tetrazolium compound [4-(3-(4-iodophenyl)-2-(4-nitrophenyl)-2H-5-tetrazolio)-1,3-benzene disulfonate) (WST-1)] to the red formazan. After exposure of colonic cells (HT-29) to fecal water for 72h, the medium is replaced with a WST-1-solution and incubated for 2h and 4h at 37°C. The intensity of the color is measured spectrophotometrically (570 nm) and is proportional to the number of living cells.

## Analysis of parameters of bone metabolism and phosphor homeostasis

The techniques for these analysis are available (mostly routine analysis and/or optimized for earlier research (f.e. FGF-23)). This part of the research project will be conducted in close corporation with Prof. Evenepoel (Laboratory of Nefrology) and Prof. Vanderschueren (Laboratory for Experimental Medicine and Endocrinology).

# STUDY PROTOCOL

The study will be performed under the direction of Prof. Dr. K. Verbeke (Laboratory Digestion & Absorption) and Prof. Dr. G. Vansant (Laboratory of Nutrition- Departement Public Health).

## Subjects

Twenty healthy subjects will be included in a randomized cross-over study with 10 subjects in each group. Group 1 will start with the high protein diet and switch to the low protein diet. In group 2 the interventions occur in reversed order (Figure 1). After an explanation of the study and its purpose written consent will be asked (‘Informed consent’: appendix 1).

Inclusion criteria:

- Healthy subjects
- Regular dietary pattern (3 meals per day)
- Age: 18-45 years
- BMI: 18.5-27 kg/m^2^

Exclusioncriteria:

- Intake of antibiotics one month prior to the study
- Abdominal surgery in the past (except appendectomy)
- Intake of medication influencing the gastrointestinal system 14 days prior to the study
- Consulting a dietician
- Severe liver- or kidney failure
- Vegetarian
- Intake of pre-/probiotics
- Participation to a study using radio-activity 1 year prior to the study

## ENDPOINTS

The primary endpoints of this study are the effects of a high, low and normal protein diet on the colonic metabolism in healthy subjects. Following parameters will be evaluated (description of used techniques, see above):

- Analysis of fat mass
- Analysis of ammonia metabolism in the colon after administration of lactose-[^15^N, ^15^N]-ureid
- Analysis of urinary p-cresol excretion as a marker of protein fermentation
- Analysis volatile organic compounds (VOC) in feces
- Analysis of genotoxicity of fecal water using the Comet Assay
- Analysis of cytotoxicity of fecal water using the WST-1 assay

The secondary endpoints of this study are the effects a high, low and normal protein diet on the phosphor homeostasis in healthy subjects. The following parameters will be evaluated (description of used techniques, see above):

- Analysis of biochemical parameters of phosphor homeostasis (serum calcium and phosphor, calcidiol, calcitriol, PTH, FGF-23)
- Analysis of parameters of bone formation and bone resorption
- Analysis of dietary phosphor and calcium
- Analysis of urinary excretion of calcium and phosphor

## DESIGN OF ADAPTED DIETS

Prof. G. Vansant will design lists with 7 breakfasts, lunches and dinners resulting in a 9% protein intake during the low protein diet. A second set of lists will be designed resulting in a 30% protein intake during the high protein diet. These lists will adapted to the energy intake of the different subjects (f.e. by leaving out or adding a slice of bread). The subjects will be able to choose a breakfast, a lunch and a dinner from these lists.

## METHODS

- On day 1 of the study subjects undergo indirect calorimetry used to measure baseline energy need. The result of this measurement and information on physical activity of the subject will be used to calculate the total energy need. These measurements will be performed by Prof. G. Vansant.
- During the first week of the study, subjects can follow their normal diet, but are asked to fill in a 7-day dietary journal.
- On day 5 of the 1^st^ week the percentage body fat is measured (impedance measurement) and a test evaluating the colonic metabolism has to be performed.
- Starting the 2^nd^ week of the study the volunteers of group 1 receive a high protein diet during week 2 and 3 of the study, while the volunteers of group 2 receive a low protein diet during week 2 and 3 of the study.
- Three days before the end of the first intervention period (=day 19) the subjects again perform a test (impedance measurement + colonic metabolism), after which they switch diet for the next 2 weeks.
- Three days before the end of the second intervention period (=day 33) the last test is performed.
- During week 3 and week 5 the volunteers again fill in a 7-day dietary record.

Subjects maintain their normal physical activity level during the entire period of the study.

On the morning of the test subjects come sober to the laboratory Digestion & Absorption. After donating a baseline urine and blood sample and measurement of the body fat percentage, a test meal is consumed by subjects. Following this test meals urinary and fecal samples have to be collected.

- Test meal:
  - Pancake supplemented with lactose-[^15^N, ^15^N]-ureid (75 mg), a capsule containing [^3^H]-PEG (185 kBq) and a glass water.
- Samples:
  - Urine samples (complete collection): 48h
  - Fecal samples (complete collection): 72h

|  | Day 0 | | Day 5 | | Day 19 | | | | | | Day 33 | | |
| --- | --- | --- | --- | --- | --- | --- | --- | --- | --- | --- | --- | --- | --- |
|  | IC | | Test 1 | | Test 2 | | | | | | Test 3 | | |
|  | |  |  |  |  | |  | |  | |  | | |
| Group 1 (n=10) | | Normal diet | | High protein diet | | | | | Low protein diet | | | |  |
|  | |  | |  |  | |  |  | |  |  | | |
| Group 2 (n=10) | | Normal diet | | Low protein diet | | | | | High protein diet | | | |  |
|  | |  | |  | |  | | |  | | |  |  |
|  | | Dietary journal | |  | | Dietary journal | | |  | | | Dietary journal |  |

IC = indirect calorimetry

Test 1-3: impedance measurement + evaluation of colonic metabolism

Figure 1: Scheme of the study and scheduled tests

## Remuneration of the volunteer

The subjects receives 100€ for participation in the study

## Dosimetry

- The stable isotope ^15^N causes no radiation and is therefore completely safe
- For determination of the oro-anal transit time the radioactive isotope ^3^H is used. The used amount of radioactivity is very low and is much less than the allowed yearly limit of intake (ALI, IRCP-68(1994)) (table 1).

Table 1: Data related to ICRP-68 (1994) standards.

| Isotope (protocol) | Internal dose (50y) (Sv/Bq) | Administered activity (Bq) | Effective dose (50y) (mSv²) |
| --- | --- | --- | --- |
| **^3^H** | 4,2 x 10^-11^ | 185 x 10^3^ | 0,007 |

# ^1^ALI=annual limit of intake

^2^Average natural background radiation in Belgium: 2.5 mSv/year (UNSCEAR 2000)

# Appendix 1: Information for the subject

**INFORMATION FOR THE SUBJECT**

| Influence of a protein diet on the colon metabolism in healthy subjects |
| --- |

AND STATEMENT OF WILLINGNESS TO PARTICIPATE

Name and surname of the subject

……………………………………………………………………………………………………….

Dear subject,

The information below describes the procedure followed during this study researching the influence of a protein diet on colon metabolism. It explains the reasons to conduct this study and contains a form on which you can confirm your willingness to participate in the study.

Background of the study

Recently, there is an increased interest in the use of high-protein/low-carbohydrate diets (protein diets) in the area of weight loss and treatment of disease. Protein intake results in increased satiety compared to other nutrients. Protein diets result in increased loss of fat in comparison with diets high in carbohydrates or low in proteins. Protein diets were also shown to reduce the risk on cardiovascular diseases. Positive effects were also found on body composition, lipid composition in the blood, glucose homeostasis or vitamin status in the body. A high protein intake is associated with increased phosphor intake, which results in changes in the phosphor homeostasis.

Goals and design of the study

Following an increased protein intake, more proteins end up in the colon. Proteins are fermented in the colon by the resident microbiota to different metabolites of which some are beneficial and others are not. Up till now no study has investigated the effects of a high protein diet on the bowel, therefore we conduct the following study studying the influence of a high dose versus a low dose of protein on the colon. We also want to investigate how high protein intake influences the phosphor homeostasis.

The study takes in total 5 weeks and consists of a run-in period of 1 week and 2 intervention periods of each 2 weeks. During one of the intervention period the diet contains a lot of protein (= on average more than normal daily protein intake in Belgium) and during the other period the diet contains less protein than normal (= on average less than normal daily protein intake in Belgium). The study has a cross-over design which means that some subjects will start with the high protein diet and switch to the low protein diet, while the other subjects will follow the diets in reverse order.

A scheme of the study and scheduled tests is given in Figure 1.

The course of the study

|  | Day 0 | | Day 5 | | Day 19 | | | | | | Day 33 | | |
| --- | --- | --- | --- | --- | --- | --- | --- | --- | --- | --- | --- | --- | --- |
|  | IC | | Test 1 | | Test 2 | | | | | | Test 3 | | |
|  | |  |  |  |  | |  | |  | |  | | |
| Group 1 (n=10) | | Normal diet | | High protein diet | | | | | Low protein diet | | | |  |
|  | |  | |  |  | |  |  | |  |  | | |
| Group 2 (n=10) | | Normal diet | | Low protein diet | | | | | High protein diet | | | |  |
|  | |  | |  | |  | | |  | | |  |  |
|  | | Dietary journal | |  | | Dietary journal | | |  | | | Dietary journal |  |

Figure 1: Study design and scheduled tests.

1. On day 1 of the study the resting energy usage will be measured using the amount of inhaled and exhaled air. During this measurement you will lay on a bed and a transparent hood will be placed over your head. This hood is used to capture and measure exhaled air. In combination with an estimation of physical activity, determined using the validated Baecke questionnaire, total energy usage will be calculated. Next, you will get an explanation on how to fill in the 7-day dietary journal.

- Eating, drinking and smoking
  - Eating and drinking are allowed until 10h prior to the measurement
  - During the 10h proceeding the measurement you are only allowed to drink water
  - Smoking is not allowed 10h prior to the measurement
- Physical activity
  - (Moderate) physical activity (f.e. riding a bike) on the morning of the measurement. Intense physical training is not allowed the evening prior to the measurement.
- Medication
  - Prescripted medication can be taken on the day of the measurement
  - A complete list of the medication should be brought to the examination. To be able to accurately interpret the results, it is important that the investigator knows which medication you use. Certain food supplements and/or homeopathic products are also important. Bringing a sample of the package is recommended.

1. During the first week of the study you are allowed to continue your normal dietary pattern and you will be asked to fill in a 7-day dietary journal. Based on your energy usage and your dietary journal individually adapted diets will be designed for the following dietary intervention periods. We will try to take your personal preferences and habits into account.

On day 5 of the first week you will perform a first test (explanation of a test, see below).

1. Starting of day 8 of the study you receive an adapted diet, which you have to follow for 2 weeks. Or you first receive a diet rich in protein, followed by a diet low in protein or vice versa. For each dietary intervention period you will receive 3 lists (one with 7 breakfasts, one with 7 lunches and one with 7 dinners) from which you can choose. These diets will contain the same amount of calories as you normal daily diet (your weight will not change), only the ratio of proteins, carbohydrates and fat will be different. Three days prior to the end of the first intervention period (= day 19) a second test will be performed.
2. Starting of day 22 (week 3) you will start with the second diet. When you followed the high protein diet during the first intervention period, you will now follow a low protein diet, and vice versa. Three days prior to the end of the first intervention period (= day 33) a second test will be performed.
3. A dietary journal has to be filled out during week 3 and week 5 of the study.

The course of a test

- On the morning of the test you come sober to the Laboratory of Digestion & Absorption
- Your body fat percentage will be determined using impedance measurement. Therefore, while laying on a bed 2 electrodes will be attached to your hands and feet.
- You donate a baseline urine sample and blood samples.
- You receive a test meal (a pancake + glass of water) as breakfast
  - The pancake contains the biomarker ^15^N to evaluate the ammonia and protein metabolism in the gut. The biomarker (^15^N) is non radioactive and therefore completely safe.
  - You will also receive a capsule with a very small amount of radioactivity (^3^H) used to measure total transit time. This product will not be absorbed in the body, but will be excreted in feces.
- Explanation of the diet and checking of the dietary journal
- Baecke questionnaire for physical activity
- You can not eat or drink 4h after consuming the pancake
- After consuming the pancake and explanation of the diet you can leave the laboratory
- After consuming the pancake the following samples need to be collected:
  - All urine for 48h
  - All feces for 72h

Remuneration

When you complete the study, you will receive a remuneration of 100€.

Risk associated with the study

Participation to the study results in no risks.

The dose of radioactivity (^3^H) is much lower than the yearly allowed limit of exposure. During participation to the study women can not be or become pregnant. To prevent pregnancy use of efficient contraceptives is necessary. A small hematoma following the blood sampling can not be excluded.

Withdrawal of participation to the study

You always have the right to refuse participation to study or to end your participation to the study. Ending your participation to the study will have no further consequences. We ask you to immediately inform the investigator when you want to end your participation. You will only receive remuneration when you completed the study.

In accordance with the Belgian law on experiments on the human person of May 7th 2004, the sponsor of the study is liable, even if faultless, for all damage which the subject and/or his rightful claimants sustained and which shows either a direct or an indirect connection with the experiment.

The sponsor has entered into an insurance contract which covers this liability. If you are caused any damage by your participation in the study, the damage will be compensated in accordance with the Belgian law on experiments on the human person of May 7th 2004.

**Informed consent:**

I have received verbal information on the above study and have read the attached written information. I have been given the chance to discuss the study and ask questions. I am completely aware of the study design and of the advantages and disadvantages associated with the study. I consent to take part in the study and I am aware my participation is entirely voluntary. I declare herewith that I will not to take part in another study during the whole duration of this study. I understand that I may withdraw at any time without this affecting my future care. By signing this information and consent form I agree to participate in this study.

Date and name of the subject Date and name of the investigator

…………………………………………. ……………………………………….

# References

^1^ Hu FB, Stampfer MJ, Manson JE et al. Dietary protein and risk of ischemic heart disease in women. Am J Clin Nutr 1999; 70: 221-227.

^2^ Noakes M, Keogh JB, Foster PR, Clifton PM. Effect of an energy-restricted, high-protein, low fat diet relative to a conventional high-carbohydrate, low-fat diet on weight loss, body composition, nutritional status, and markers of cardiovascular health in obese women. Am J Clin Nutr 2005; 81: 1298-1306.

^3^ Cummings JH. Short chain fatty acids in the human colon. Gut 1981; 22: 763-779.

^4^ Smith EA, Macfarlane GT. Enumeration of amino acid fermenting bacteria in the human large intestine: effects of pH and starch on peptide metabolism and dissimilation of amino acids. FEMS Microbiol Ecol 1998; 25: 355-368.

^5^ Fooks LJ, Fuller R, Gibson GR. Prebiotics, probiotics and human gut microbiology. Int Dairy J 1999; 9: 53-61.
